# Position Statement of the Polish Academy of Sciences’ Committee of Human Nutrition Science on the Principles for the Nutrition of Preschool Children (4–6 Years of Age) and Early School-Age Children (7–9 Years of Age)

**DOI:** 10.34763/jmotherandchild.20232701.d-23-00094

**Published:** 2023-12-31

**Authors:** Halina Weker, Mariola Friedrich, Katarzyna Zabłocka-Słowińska, Joanna Sadowska, Anna Długosz, Jadwiga Hamułka, Jadwiga Charzewska, Piotr Socha, Lidia Wądołowska

**Affiliations:** Department of Nutrition, Institute of Mother and Child, Warsaw, Poland; Department of Applied Microbiology and Human Nutrition Physiology, Faculty of Food Sciences and Fisheries, West Pomeranianu University of Technology, Szczecin, Poland; Institute of Health Sciences, University of Opole, Opole, Poland; Faculty of Chemical Technology and Engineering, University of Science and Technology, Bydgoszcz, Poland; Department of Human Nutrition, Institute of Human Nutrition Sciences, Warsaw University of Life Sciences (SGGW-WULS), Warsaw, Poland; National Institute of Public Health NIH – National Research Institute, Warsaw, Poland; Department of Gastroenterology, Hepatology, Eating Disorders and Paediatrics, Institute ‘Monument - Children’s Health Center’, Warsaw, Poland; Department of Human Nutrition, University of Warmia and Mazury in Olsztyn, Poland; WSB University in Wroclaw, Wrocław, Poland

The position statement of the Committee on Human Nutrition Science, Polish Academy of Sciences is based on guidelines and positions drafted by scientific societies, reputable institutions and teams of experts, including the WHO 1World Health Organization., ESPGHAN 2European Society for Paediatric Gastroenterology Hepatology and Nutrition., AAP 3American Academy of Pediatrics., EFSA 4European Food Safety Authority. and the National Center for Nutrition Education in Poland.

With respect to the aforementioned documents and the possibility of their adaptation in Polish conditions, as well as on the basis of a review of the current literature on nutrition of preschool and early school-age children and the results of scientific research in this area conducted in Poland, the experts (members of the Task Force for Children and Adolescents Nutrition of the Polish Academy of Sciences’ Committee on Human Nutrition Science) developed nutritional recommendations for preschool and early school-age children.

The position statement along with its justification is presented below.

## Principles for the nutrition of preschool children (4–6 years of age) and early school-age children (7–9 years of age)

1. **Proper nutrition**, including adequate dietary intake of energy and nutrients, **determines children’s optimal psychosomatic development and their health, both in childhood and in later life.** Therefore, continuous control of the nutrition of children by their parents and/or caregivers is necessary.
2. **Adherence to a model of safe nutrition of children**, with its basis being the proper organisation of meals and a varied selection of foods with respect to the nutritional profile of the diet, **has a beneficial impact on growth, development and health status**.
  – **Proper organisation of meals** eaten at home/kindergarten/school promotes appropriate eating habits and behaviours in children. A daily diet should consist of four to five meals, including three main meals (breakfast, lunch, dinner). Other solutions concerning the organisation of nutrition of preschool and early school-age children should be adjusted to individual needs of the children.
  – **Selection of food products, including beverages, is important to balance out the diet of children** with respect to their energy needs and nutrient requirements. **In the diet, 10–20% of total energy should be provided by protein, 20–35% should come from fats** (of which <10% of total energy from saturated fatty acids and <1% of total energy from trans isomers of polyunsaturated fatty acids and the remaining energy from other unsaturated fatty acids) and **45–65% of total energy intake should be provided by carbohydrates**, mainly complex carbohydrates, with reduced intake of free sugars 5Free sugars are sugars added to food and sugars naturally present in honey, syrups, fruit juices and juice concentrates, excluding fructose present in fruits and lactose present in dairy products. (up to <10% of energy, with the recommendation to further reduce their intake to less than 5% of energy).
  – **It is recommended that the nutrient profile of children’s diets should be compliant with the current nutrition standards.** In the nutrition of children, attention should be paid to the **potential risk of deficiency of calcium, iron and vitamin D, and to adequate intake of polyunsaturated fatty acids (PUFA)**. Vitamin D intake should comply with medical recommendations.
3. **Monitoring of nutritional status of children by systematically measuring their body weight and height** constitutes the basis for the assessment of their development and for early prevention of the risk of being underweight or overweight, and the risk of other non-communicable chronic diseases, including those related to obesity.
4. **Nutrition of children in kindergartens/schools** should comply with the recommendations, including those taking into account the risks and benefits associated with vegetarian diets in children (*Position Statement* 6 *of the Committee on Human Nutrition Science, 2019*) and **requires special attention in the following areas:**
  – **Variety of the diet,** to ensure a large variety of foods consumed by children;
  – **Increased intake of vegetables and fruits,** among others through self-selection of the types and amounts of vegetables and fruits from the provided range of vegetables and fruits;
  – **Selection of products,** including seasonal and local products, for composition of meals;
  – **Availability of vegetarian meals and specialist diets,** if it is necessary to adhere to them; and
  – **Avoiding food waste,** among others by offering children the possibility to select the size of the portion or to take the uneaten food home in their own boxes.
5. **Physical activity of children and care for their health** (e.g. proper hygiene, including of oral cavity, sleep hygiene) are necessary **as an important element of preventive care**.
6. **Health education**, including nutrition education of children, extending also to their families and kindergarten/school environment, **should be systematic and treated as a long-term investment in the health of the society**.

## Model food rations for preschool (4–6 years of age) and early school-age (7–9 years of age) children

Tables 1 and 2 present model food rations, that is, amounts of products from various groups expressed in grams that are recommended for daily intake, and the number of portions, while Table 3 presents the approximate size of one portion for children, which may be helpful in planning the diets of children.

**Table 1.** Model food rations expressed in products for children aged 4–6 years, recommended by the PAS Committee on Human Nutrition Science and other teams of experts

	**Product groups**		**The amount of products in daily diet according to various expert groups**					

			**Polish recommendations**				**American recommendations, 2020 (per 1400 kcal)^2^**	

			**Unit**	**PAS Committee on Human Nutrition Science 2023 (per 1400 kcal/day)**	**Turlejska et al. 2004^3^**	**IŻŻ 2001^4^**	**American measures [cup/day or ounce / day]**	**Converted to grams [g/day]**
**I. Starch products**	**1**	**Grain products and potatoes**		-	-	-	Wholegrain products 2.5 ounces	70
							Refined grain products 2.5 ounces	70
		Bread – wheat, rye, mixed	g/day	**120**	170	150		
		Flour, pasta	g/day	**30**	50	30		
		Groats, rice, breakfast cereal	g/day	**30**	30	35		
	**1A**	**Potatoes**	g/day	**100**	200	200		

**II. Vegetables and fruits**	**2**	**Vegetables and fruits**	g/day	**-**	-	650		
		Vegetables	g/day	**300**	400	400	1.5 cup	112
		Fruits	g/day	**200**	250	250	1.5 cup	225
	**2A**	**Pulses, nuts, other seeds**	g/day	**5**	10 (pulses/nuts)	-	Nuts/seeds/soybean products 0.5 ounce	14

**III. Protein products**	**3**	**Milk and dairy products**		**-**	-	-	Milk and dairy products 2.5 cups	625
		Milk/fermented milk beverages	g/day	**400/100**	550	550		
		Fresh/cottage cheeses	g/day	**30**	60	45		
		Rennet cheeses	g/day	**10**	10	5		
	**4**	**Meat, cold meats, fish, eggs**		**-**	-	-	Meat, poultry, eggs 2.7 ounces	76
		Meat^1^, poultry^1^, cold meats	g/day	**50**	Meat/poultry^1^ 40 Cold meats 20	Meat/poultry^1^ 30 Cold meats 20		
		Fish^1^	g/day	**20**	20	5	0.9 ounce	25
		Eggs	pcs/day	**½**	¾	¾		

**IV. Fats and other**	**5**	**Fats**	g/day	**-**	-	38		
		Animal fats: butter and cream	g/day	**10**	25	25		
		Vegetables fats: oils	g/day	**10**	Oils/margarines 12 Mixed fats 2	13	Vegetable oils 17 g	17
	**6**	**Sugar and sweets**	g/day	**Not more than 10^5^**	35	30		
	**7**	**Other**		-			90 kcal/day	

1 Meat without bones, fish – fillets

2 *Dietary Guidelines for Americans, 2020–2025. 9^th^ Edition. 2020. U.S. Department of Agriculture and U.S. Department of Health and Human Services.*

3 *Turlejska H., Pelzner U., Szponar L., Konecka Matyjek E. Zasady racjonalnego żywienia – zalecane racje pokarmowe dla wybranych grup ludności w zakładach żywienia zbiorowego [Principles of rational nutrition – recommended food rations for selected groups of the population in mass catering establishments]. Wyd. Ośrodka Doradztwa i Doskonalenia Kadr Sp. z o.o., Gdańsk 2004.*

4 *Dzieniszewski J, Szponar L, Szczygieł B, Socha J. Podstawy naukowe żywienia w szpitalach [Scientific basis for nutrition in hospitals], IŻŻ Warsaw 2001.* Comment: model daily food rations were developed for hospital patients.

5 The smaller quantity, the better.

Explanations of American measures: Fruits: 1 cup = 150 g; Vegetables: 1 cup = 75 g; Solid/liquid products: 1 ounce = 28 g/30 ml; Liquids for drinking: 1 cup = 250 ml

**Table 2.** Model food rations expressed in products for children aged 7–9 years, recommended by the PAS Committee on Human Nutrition Science and other teams of experts

		**Product groups**	**The amount of products in daily diet according to various expert groups**					

			**Polish recommendations**				**American recommendations, 2020 (per 1800 kcal)^2^**	

			**Unit**	**PAS Committee on Human Nutrition Science 2023 (per 1400 kcal/day)**	**Turlejska et al. 2004^3^**	**IZZ 2001^4^**	**American measures [cup/day or ounce /day]**	**Converted to grams [g/day]**
**I. Starch products**	**1**	**Grain products and potatoes**		-	-	-	Wholegrain products 3 ounces	85
							Refined grain products 3 ounces	85
		Bread – wheat, rye, mixed	g/day	**150**	210	200		
		Flour, pasta	g/day	**40**	60	40		
		Groats, rice, breakfast cereal	g/day	**35**	35	35		
	**1A**	**Potatoes**	g/day	**120**	250	250		

**II. Vegetables and fruits**	**2**	**Vegetables and fruits**	g/day	-	-	730		
		Vegetables	g/day	**350**	430	430	2.5 cups	190
		Fruits	g/day	**250**	300	300	1.5 cup	225
	**2A**	**Pulses, nuts, other seeds**	g/day	**10**	12 (pulses/nuts)	-	Nuts/seeds/soybean products 0.6 ounce	17

**III. Protein products**	**3**	**Milk and dairy products**		-	-	-	Milk and dairy products 2.5 cups	625
		Milk/fermented milk beverages	g/day	**350/150**	550	500		
		Fresh/cottage cheeses	g/day	**50**	65	50		
		Rennet cheeses	g/day	**15**	12	10		
	**4**	**Meat, cold meats, fish, eggs**		-	-	-	Meat, poultry, eggs 3.3 ounces	90
		Meat^1^, poultry^1^, cold meats	g/day	**70**	Meat/poultry^1^ 40 Cold meats 20	Meat/poultry^1^ 30 Cold meats 20		
		Fish^1^	g/day	**20**	20	10	1.1 ounce	30
		Eggs	pcs/day	**½**	½	½		

**IV. Fats and other**	**5**	**Fats**	g/day	-	-	50		
		Animal fats: butter and cream	g/day	**20**	27	33		
		Vegetable fats: oils	g/day	**10**	Oils/margarines 21 Mixed fats 2	17	Vegetable oils 22 g	22
	**6**	**Sugar and sweets**	g/day	**Not more than 10^5^**	45	40		
	**7**	**Other**		-	-	-	190 kcal/day	

1 Meat without bones, fish – fillets

2 *Dietary Guidelines for Americans, 2020–2025. 9^th^ Edition. 2020. U.S. Department of Agriculture and U.S. Department of Health and Human Services.*

3 *Turlejska H., Pelzner U., Szponar L., Konecka Matyjek E. Zasady racjonalnego żywienia – zalecane racje pokarmowe dla wybranych grup ludności w zakładach żywienia zbiorowego [Principles of rational nutrition – recommended food rations for selected groups of the population in mass catering establishments]. Wyd. Ośrodka Doradztwa i Doskonalenia Kadr Sp. z o.o., Gdańsk 2004.*

4 *Dzieniszewski J, Szponar L, Szczygieł B, Socha J. Podstawy naukowe żywienia w szpitalach [Scientific basis for nutrition in hospitals], IŻŻ Warsaw 2001.* Comment: model daily food rations were developed for hospital patients.

5 The smaller quantity, the better.

Explanations of American measures: Fruits: 1 cup = 150 g; Vegetables: 1 cup = 75 g; Solid/liquid products: 1 ounce = 28 g/30 ml; Liquids for drinking: 1 cup = 250 ml

**Table 3.** Approximate size of portions of selected products and number of portions recommended for daily consumption by children aged 4–6 and 7–9 years

**Product groups**	**Number of portions/day for children aged**		**Approximate size of 1 portion - examples for children**

	**4–6 years**	**7–9 years**	
**Starch products,** including	**5**	**5–6**	
• grain products	**4**	**4–5**	2–3 slices of wheat-rye bread (3 x 35 g) 1–2 wholemeal bread rolls 1 wholegrain bread roll (50 g) ½-¾ glass of cooked pasta ½ glass of cooked groats, e.g. buckwheat groats, barley groats, rice, cereal
• potatoes	**1**	**1–2**	• 1 large or 2 small potatoes
**Vegetables**	**5**	**5**	yellow vegetables, e.g. ½ glass of French beans or corn orange vegetables, e.g. ½ glass of grated carrot white vegetables, e.g. ½ glass of cut cabbage, 5 white asparagus, 5–6 cauliflower florets red vegetables, e.g. 1 tomato, ½ red pepper green vegetables, e.g. a handful of rocket, spinach, 2–3 iceberg lettuce leaves, fresh or pickled cucumber
**Fruits**	**3**	**3**	1 medium apple 1 medium banana 1 pear 5-6 plums 7-8 strawberries 1 mandarin orange ½ glass of raspberries, currants or blueberries
**Pulses, nuts, other seeds**	**1**	**1–2**	1 teaspoon of lentil paste 1 teaspoon of sunflower seeds 2 walnuts without shell 4 almonds
**Protein products,** including:	**4**	**4–5**	
• milk and dairy products	2	2	1 glass of milk ½-1 cup of yoghurt/kefir/buttermilk 2–3 tablespoons of cottage cheese a slice of rennet cheese
• meat, cold meats, poultry, fish, eggs	2	2–3	a slice of baked pork loin, tenderloin ½ slice of lean ham 1 small poultry meatball 1 tablespoon of goulash, e.g. of veal 1 fish fillet (e.g. cod, salmon, blue grenadier) 1 egg
**Fats**	**3–4**	**5–6**	1 teaspoon of butter 1 teaspoon of cream 1 teaspoon of olive oil 1 teaspoon rapeseed oil

## Justification

The World Health Organization defines health as a state of complete physical, mental and social well-being and not merely the absence of disease or infirmity. The health status of a child, including its psychosomatic development from as early as prenatal period and infancy, is related to its nutrition [48, 14, 74, 29, 117]. Proper diet of children at preschool and early school age also influences their optimal nutritional status. This is confirmed by the results of research conducted in numerous research centres around the world, also in Poland [46, 109, 57, 16, 110, 29, 137, 80, 115]. A national study conducted in the years 2017–2020 on comprehensive assessment of nutrition and nutritional status of preschool and school-age children (6–18 years of age) found that two-thirds of the subjects had a proper body weight, 29.1% were overweight, while 2.2% underweight. Although no direct negative impact of the three identified dietary patterns (“Healthy”, “Sweet-Western”, “Traditional”) on overweight, obesity or underweight in children was found, from among the analysed nutritional factors, the duration of breastfeeding had an important protective impact with respect to excessive body weight in school-age children [57].

Compared to earlier developmental stages, that is, infancy and post-infancy, the period between 4 and 6 years of age, defined as preschool age, and between 7 and 9 years of age, defined as early school age, are characterised by a slower growth rate, but intensive mental development, in the cognitive, emotional, social and motoric spheres [30, 134]. The principles for the nutrition of preschool and early school-age children with respect to the specificity of their development in those age ranges are presented below.

## 1. Selected factors/issues and health status of preschool and early school-age children

### 1.1 Developmental processes of preschool and early school-age children determining their nutrition

It is difficult to rigorously specify the limits of the development of preschool (4–6 years of age) and early school-age (7–9 years of age) children. Similar to earlier developmental periods, in this period, there is a great inter-individual variation, while the differences in body weight and height, their proportions, and the maturity of individual organs and systems are more pronounced than in post-infancy. This is probably related to the production of human growth hormones (HGH – somatotropin) by children. The hormone secretion increases in the first 4 years of life and amounts to approximately 90 μg per day in children before sexual maturity. HGH secretion by the cells of the anterior lobe of the pituitary gland is strongly stimulated by ghrelin, which acts synergistically with GHRH (somatoliberin) and the release of which may be inhibited by excessive activity and exhaustion of the child [140, 141]. The maximum release of HGH takes place at night, 1 to 4 hours after falling asleep [94, 2]. HGH acts among others by stimulating the secretion of the so-called insulin-like growth factors I and II (IGF-I and IGF-II) by the liver but also by other tissues. HGH, along with IGF-I, stimulates protein synthesis in the body, but also participates in the metabolism of carbohydrates, lipids and minerals [19, 77]. Under its influence, during the growth of the body, the metaphyseal cartilages of long bones widen, and the bones grow in length [78]. Therefore, the time and duration of sleep of children, as well as the children’s diet, in particular the type and amount of protein in the diet, are also important during the developmental period [51]. Between 5 and 6 years of age, fast increase in muscle mass is observed, which results in/enables high mobility of children, and thus special attention must be paid to the energy value of the diet. However, compared to high dynamics of the processes taking part in the bodies of infants and post-infantile children, physical development of children aged 4–6 years and 7–9 years is slower and changes take place gradually. Annual increases in body height amount on average to 6–8 cm and 4.5–5.5 cm, respectively, for preschool and early school-age children, and in body weight to 2–3 kg. Boys grow more intensively between 3 and 6 years of age, and girls between 3 and 4 years of age [27].

During the discussed developmental periods, a particularly intense development of cognitive functions, including speech, memory, thinking and attention, takes place [136]. It is related to further development of the nervous system, including the formation of new neurons, axon myelination, glial cell proliferation and neurotransmitter biosynthesis. This requires special attention to ensure an adequate dietary intake of protein with high nutritional value (containing, among others, amino acids such as tryptophan, phenylalanine, tyrosine), polyunsaturated fatty acids from the n-3 family (including docosahexaenoic acid, DHA and eicosapentaenoic acid, EPA), specific minerals (such as iron, iodine, zinc), vitamins from B group (B1, B6, PP, B12, folic acid) and vitamin D.

During the preschool period, children’s appetites may change day by day, which parents/caregivers should have in mind in particular when feeding. During preschool and early school age, changes occur in children’s digestive tracts, which, although it has full digestive efficiency, is very sensitive to various factors, including an improper diet and foods of inappropriate health quality (including microbiological quality). The review of literature demonstrates that in preschool and early school-age children, despite the functional maturation of their kidneys [108], attention still should be paid to dietary sodium intake due to the possibility of hypertension in adulthood [42, 60]. This is also the period when children begin to lose their primary teeth and first permanent teeth appear, which also requires changes to the diet [47].

### 1.2 Nutritional status of children and the risk of nutrition-related non-communicable diseases

Numerous irregularities observed in the nutrition of children have plenty of consequences, including with respect to health. The effects of nutritional irregularities in children are not easy to identify since, most often, they are manifested as subclinical symptoms of nutrient deficiencies of a non-specific nature and mild course and as symptoms of inappropriate energy intake (being underweight or overweight). Malnutrition in every form presents significant threats to health, in particular of young children. Currently, we see a double burden of malnutrition that includes both energy and nutrient deficiency and the resulting underweight and stunting in children, as well as deficiency of numerous nutrients, also in some overweight children. Undernutrition, including deficiencies of vitamins and minerals, and deficiencies of bioactive compounds, impairs healthy, regular development and well-being. At the same time, growing rates of overweight and obesity are linked to a rise in non-communicable chronic diseases. According to the WHO, both underweight and overweight are among ten factors which are most harmful to human health [128, 131, 72].

Overweight and obesity are not only aesthetic issues and developmental disorders, but also have long-term health consequences. The metabolic consequences of obesity, resulting from both the amount of adipose tissue in the body and the inflammatory processes occurring therein, include carbohydrate metabolism disorders, which include prediabetes and type 2 diabetes; lipid metabolism disorders; hypertension; non-alcoholic fatty liver disease; hormonal disorders and skin lesions. Obesity is also associated with gastroesophageal reflux, lung ventilation disorders (obstructive sleep apnea, asthma), orthopaedic complications, but also with mental problems, for example, poor well-being, lack of acceptance of one’s own body, lowered self-esteem, social isolation and thus difficult relationships with other people. This may lead to neurotic disorders or depression and eating disorders, which also have serious health consequences.

In view of numerous threats associated with inappropriate lifestyle, including both excessive body weight and underweight, and their impact on health, it is necessary to constantly monitor the physical development of children using anthropometric measurements.

The diagnosis of overweight and obesity in children and adolescents is more complex than in adults. It is necessary to take into account the specifics of the developmental period, the course of growth and maturation depending on gender, as well as the adipose tissue distribution. Individual differences in the structure and composition of children’s bodies are also important. Therefore, regular anthropometric measurements are necessary (at least once a year), and the results obtained should be compared with appropriate reference systems taking into account children’s age and gender, that is, development standards, developmental norms and reference values [101].

The diagnosis of overweight and underweight in children is most often performed using anthropometric measurements, such as height, body weight, waist and hip circumference, and indicators calculated on their basis, including the body mass index (BMI) and the waist-to-height ratio (WHtR). In order to interpret the results for children over 4 years of age, using OLA/OLAF percentile charts is recommended, and in the case of BMI, using BMI percentile charts and z-score developed by the WHO (WHO Child Growth Standards for children 0–5 years), while in children over 5 years of age, using BMI percentile charts for the Polish population (OLA/OLAF) is recommended [101, 58, 102]. It is emphasized that BMI values in children aged 1 and 5 years may be important predictors of body weight in the future [12]. Waist circumference, after reference to appropriate developmental norms (OLA/OLAF percentile charts), facilitates the assessment of the adipose tissue distribution in the body, and the proposed criterion for diagnosing obesity is defined as values above the 95th percentile [11]. WHtR values of ≥ 0,5 may be useful for finding abdominal obesity in individuals starting from 5 years of age [8].

The most common childhood diseases resulting from dietary errors and other lifestyle irregularities, apart from those mentioned above, also include distortions of the development of the skeletal system and teeth.

### 1.3 Oral health

Oral health is a constituent of overall health and is largely determined by health behaviours of an individual and by environmental factors (sociodemographic, economic, cultural) [96]. Oral health in children means the lack of caries in primary and permanent teeth, the appropriate condition of periodontal tissues and the absence of dental abnormalities and malocclusion [68]. The oral health of children affects their quality of life and may be the reason for problems with chewing, swallowing, breathing, and sleeping, as well as a source of psychosocial problems for children and their families [17].

In Poland, the major health problems in terms of oral health in children during early childhood include high prevalence and intensity of caries in primary teeth, negligence with respect to prevention and treatment, high incidence and severity of complications of untreated dental caries, high prevalence of malocclusions and dental anomalies [84, 83, 87, 86, 54]. Prevalence and intensity of dental caries in children and adolescents in Poland are among the highest in Europe. Carious lesions are reported in over 40% of 3-year-olds, and the incidence of caries increases with age. Dental caries is present in 76.8% 5-year-olds, 81.6% of 6-year-olds, and in children at the age of 7 its incidence stands at 85.1% [90]. In a 5-year-old almost five teeth on average are affected by caries. In Poland, 13.2% of children aged 5 years have never visited a dentist. The conservative dentistry treatment ratio among 5-year-old children is at a very low level and amounts to 0.15 7The conservative dentistry treatment ratio is the quotient of the number of teeth with fillings and the sum of the number of teeth with active caries and fillings. [87, 84]. Epidemiological research shows that malocclusions occur in 40–70% of Polish preschool children [54].

The formation of proper hygiene and nutritional habits and health-promoting attitudes in children, and, consequently, subsequent oral health of the children are influenced primarily by parents’ health awareness of the principles of proper oral hygiene and the importance of undisturbed physiological activities such as breathing, swallowing, chewing and speaking, as well as their activity in eliminating harmful eating habits and using fluoride prevention [113, 49]. According to the recommendations of a team of experts in the field of paediatric dentistry and paediatrics, established as part of the activities of the Working Group on Fluoride Prevention of the Polish Branch of the Alliance for a Cavity-Free Future (ACFF), in children under 6 years of age the only form of home prevention is brushing the teeth twice a day using a fluoride toothpaste (concentration 1000 ppm F): in the morning after breakfast and in the evening after a meal. Children up to the age of 8 years should have their teeth brushed by their parents/caregivers, and in the following year, this procedure should be supervised. Fluoride prophylaxis, apart from a proper diet, remains the basic method of preventing dental caries [53, 89, 85, 88].

## 2. Nutrition of preschool and early school-age children

### 2.1 Basics of nutrition – recommendations, standards

Proper nutrition is one of the most important elements of a healthy lifestyle that affects human health and well-being from the moment of conception, through childhood, adulthood, and old age. Food should be safe, not only in terms of broadly understood health quality, including microbiological and technological purity but mainly in terms of nutritional value. Appropriate quality of food determines good health and the achievement of potentially the highest quality of life in physical, emotional and intellectual terms [37, 51, 131].

Proper feeding fulfils all nutritional needs of children in terms of energy and nutrients, except for vitamin D, which in our latitude should be administered as a dietary supplement/drug in accordance with medical recommendations [97, 120]. Monitoring the diet with respect to the nutritional status, which is the result of nutrient intake, digestion, absorption and assimilation, allows for early intervention if potential disturbances in the growth and maturation processes are found [138, 40, 9, 34, 1, 6, 10, 71, 22, 104].

The nutrition of children should comply with the model of safe nutrition in which special attention is paid to the organisation/frequency of meals served to children within a day, selection of products in the diet and the nutrient profile, as well as other factors, including those promoting healthy behaviours and eating habits [82, 126, 127].

The most important components of the safe nutrition model are presented below:

(1) The recommended number of meals to be consumed by preschool and early school-age children in a day should be four to five. The meals can be prepared and served at home and/or in care and educational facilities offering mass catering (e.g. kindergartens, children’s homes, schools). The solutions concerning the organisation of the feeding of preschool and school children depend on the form of care. Over 90% of children aged 3–6 years in Poland attend kindergartens [93].

Depending on the time spent by children in kindergartens, they have from one to three meals outside the home. Those meals usually include breakfast and/or lunch, and afternoon snack, while at home children usually eat dinner or lunch and dinner in one. Early school-age children usually have meals at home (breakfast, lunch, dinner). However, approximately 17–19% of children aged 8 years do not eat breakfast at all [35, 36], and a significant percentage of children do not bring second breakfast with them and do not eat any meal at school [57, 122, 73].

(2) According to the guidelines of national and international, including European, scientific societies, special attention should be paid to the selection of foods in the diets of children, when the meals are composed [119, 30, 103]. Vegetables and fruits (fresh, frozen), grain products and protein products should constitute the basis of the diet (Tables 1, 2, 3).
(3) A balanced diet consisting of high-quality food must have an appropriate energy value. The energy value of the diet for children aged 4–6 years with moderate physical activity amounts to 1400 kcal (5.8 MJ/day), while for children aged 7–9 years with moderate physical activity to 1800 kcal (7.4 MJ/day). The nutrient profile, that is, intake of macronutrients: protein, fat and carbohydrates, as well as vitamins and minerals, should comply with nutritional standards [51]. Nutritional standards are periodically updated in line with the current results of objectivised research.

Table 4 presents the current nutritional standards developed by the team from the National Institute of Public Health – National Institute of Hygiene – National Research Institute (NIZP-PZH-PIB) for the Polish population, including preschool and school-age children [51], in terms of the adequate intake (AI) or Estimated Average Requirement (EAR) and Recommended Dietary Allowances (RDA) [51].

**Table 4.** Nutritional standards for children aged 4–6 and 7–9 years [Jarosz M et al. 2020] [51]

**Energy**							
Age [years]	Body weight [kg]	Energy [MJ/day]			Energy [kcal/day]		

		Physical activity [PAL]			Physical activity [PAL]		

		Low	Moderate	High	Low	Moderate	High
4–6	19	-	**5.8** (PAL: 1.5)	-	-	**1400** (PAL: 1.5)	-
7–9	27	**6.3** (PAL: 1.35)	**7.4** (PAL: 1.6)	**8.6** (PAL: 1.85)	**1550** (PAL: 1.35)	**1800** (PAL: 1.6)	**2100** (PAL: 1.85)

**Protein**					
Age [years]	Body weight [kg]	EAR		RDA	

		Protein in the national food ration		Protein in the national food ration	

		g/kg of body weight /day	g/person/day	g/kg of body weight /day	g/person/day
4–6	19	**0.84**	**16**	**1.10**	**21**
7–9	27	**0.84**	**23**	**1.10**	**30**

**Fat**				
Age [years]	Body weight [kg]	RI for fat [g/person/day] with various shares of energy from fats		

		20 [% of energy]	30 [% of energy]	35 [% of energy]
4–6	19	**31** (PAL: 1.5)	**47** (PAL: 1.5)	**54** (PAL: 1.5)
7–9	27	**34/40/47** (PAL: 1.35/1.6/1.85)	**52/60/70** (PAL: 1.35/1.6/1.85)	**60/70/82** (PAL: 1.35/1.6/1.85)

**Carbohydrates and dietary fibre**		
Age [years]	RI for carbohydrates for individuals over 1 year of age [% of energy]	AI for dietary fibre [g/day]
4–6	**45–65**	**14**
7–9	**45–65**	**16**

**Vitamins**					
Age [years]	Vitamin A [µg]		Vitamin D [µg]	Vitamin E [mg]	Vitamin K [µg]

	EAR	RDA	AI	AI	AI
4–6	**300**	**450**	**15**	**6**	**20**
7–9	**350**	**500**	**15**	**7**	**25**

**Vitamins cont.**								
Age [years]	Vitamin B_1_ [mg]		Vitamin B_2_ [mg]		Vitamin PP [mg]		Choline [mg]	Pantothenic acid [mg]

	**EAR**	**RDA**	**EAR**	**RDA**	**EAR**	**RDA**	**AI**	**AI**
4–6	**0.5**	**0.6**	**0.5**	**0.6**	**6**	**8**	**250**	**4**
7–9	**0.7**	**0.9**	**0.8**	**0.9**	**9**	**12**	**250**	**4**

**Vitamins cont.**									
Age [years]	Vitamin B_6_ [mg]		Biotin [µg]	Vitamin B_12_ [µg]		Vitamin C [mg]		Folic acid [µg]	

	EAR	RDA	AI	EAR	RDA	EAR	RDA	EAR	RDA
4–6	**0.5**	**0.6**	**12**	**1.0**	**1.2**	**40**	**50**	**160**	**200**
7–9	**0.8**	**1.0**	**20**	**1.5**	**1.8**	**40**	**50**	**250**	**300**

**Minerals**												
Age [years]	Calcium [mg]		Phosphorus [mg]		Magnesium [mg]		Iron [mg]		Zinc [mg]		Copper [mg]	

	EAR	RDA	EAR	RDA	EAR	RDA	EAR	RDA	EAR	RDA	EAR	RDA
4–6	**800**	**1000**	**410**	**500**	**110**	**130**	**4**	**10**	**4**	**5**	**0.3**	**0.4**
7–9	**800**	**1000**	**500**	**600**	**110**	**130**	**4**	**10**	**4**	**5**	**0.5**	**0.7**

**Minerals cont.**								
Age [years]	Iodine [µg]		Selenium [µg]		Fluorine [mg]	Sodium [mg]	Potassium [mg]	Chlorine [mg]

	EAR	RDA	EAR	RDA	AI	AI	AI	AI
4–6	**65**	**90**	**23**	**30**	**1.0**	**1000**	**1100**	**1550**
7–9	**70**	**100**	**23**	**30**	**1.2**	**1200**	**1800**	**1850**

*Explanations:*

* Guidelines for vitamin D according to the US Institute of Medicine. The current recommendations for prevention and treatment of vitamin D deficiency call for supplementation with cholecalciferol in the dose of 600–1000 IU/day (15–25 μg/day) in children aged 4–10 years, with reference to body weight and vitamin D intake from the diet [Pludowski P et al. 2023] [98].

PAL - Physical Activity Level

EAR - Estimated Average Requirement

RDA - Recommended Dietary Allowances

AI - Adequate Intake

RI - Reference Intake

– **Proteins** are mainly used for building the cell structures in the body. They also act as apoproteins in oxygen carriers (globin), control the growth of cells and their vital functions and receive, transmit and store information in the nervous system. Amino acids, that is, the basic components of proteins, are necessary for the synthesis of many important compounds necessary to maintain the functioning of the body, for example, nucleic acids, enzymes, hormones and some vitamins.

The sources of complete proteins in children’s diets include meat and meat products, milk and dairy products, including fermented milk products, and eggs. Plant protein derived mainly from grain products and pulses should complement the range of amino acids provided with animal protein. Thus, the recommended ratio of animal protein to plant protein is 2:1. Excessive protein intake by children may be related to the risk of overweight and obesity [125].

– The role of **fats** in the body is not limited only to their energy function (1 g of fat provides 9 kcal), but they also have a structural function. The components of fats, mainly fatty acids, and in particular polyunsaturated fatty acids, are structural elements of cell membranes, including the brain, and precursors of biologically active substances, that is, hormones, enzymes and vitamins [37]. Therefore, the type and quality of consumed fats are very important. In the diets of children, it is important to ensure an appropriate share of vegetable oils, including rapeseed oil, olive oil, fatty saltwater or freshwater fish, as well as milk fat (butter). The insufficient amount of fat in children’s diets increases the risk of stunting and even malnutrition. However, excessive intake of products containing saturated fatty acids and cholesterol (e.g. fatty meat, high-fat dairy products) and foods containing trans isomers of polyunsaturated fatty acids (e.g. confectionery) increase the risk of obesity, cardiovascular diseases and metabolic disorders [30, 31, 65]. However, there are studies which prove that the consumption of milk and dairy products with a higher fat content by children is not associated with excessive weight gain and, consequently, is not associated with overweight and obesity in adulthood [50].

– **Carbohydrates** mainly provide energy (1 g provides 4 kcal) to the body. In the diets of children, complex carbohydrates (starch) from grain products, vegetables and pulses should prevail. Starch is present in groats, flour and products/dishes prepared from flour and cereals, as well as tuber vegetables (e.g. potatoes, sweet potatoes). A very important role, including the regulation of the digestive tract functioning, is played by dietary fibre, which is present in whole grain products and vegetables, and, in smaller amounts, in fruits [28, 117, 15].

Excessive share of carbohydrates, in particular simple sugars (e.g. glucose, fructose) and disaccharides (e.g. sucrose), in the diet is unfavourable for children’s health, because it may promote increased weight gain, leading to overweight or obesity and other non-communicable chronic diseases (e.g. diabetes, cardiovascular diseases) and dental caries [46, 30, 15]. The recommended share of total carbohydrates in the diet is the result of the percentage of energy that should be provided after subtracting the energy provided by protein (10–20% of total daily energy) and fat (20–35% of total daily energy), that is, it is approximately 45–65% of the total daily energy intake.

In general, the recommendations of various expert bodies on sugar consumption are consistent – the less sugar in children’s diet, the better for their health – but they refer to different definitions of sugars 8Sugars – the term is currently applied to mono- and disaccharides present in foods (regardless of their origin, i.e. natural or added). Sugars do not include polyols (alcohols - derivatives of monosaccharides, e.g. sorbitol, xylitol, mannitol), although according to European food law they are classified as carbohydrates [37]., for example, added sugars 9Added sugars mean sugars (among others sucrose, fructose, glucose, starch hydrolysates, e.g. glucose syrup and highly fructose syrup) added during the manufacturing food or preparing food for consumption (by the manufacturer, cook or consumer). The term was introduced to differentiate from carbohydrates naturally present in food. The main sources of added sugars include white and brown sugar, melass, corn and maple syrup, honey, dextrose [37]. (older recommendations) or free sugars 10Free sugars are sugars added to food and sugars naturally present in honey, syrups, fruit juices and fruit juice concentrates. They do not include fructose present in fruits and lactose present in dairy products [37, 132]. (newer recommendations). According to the WHO [132], free sugar intake should be limited to less than 10% of total energy intake (a strong recommendation), with the suggested even more radical reduction of the free sugar intake to less than 5% of the total energy intake (a conditional recommendation). According to the Polish Society for Paediatrics Gastroenterology, Hepatology and Nutrition, in children from 2 years of age and in adolescents, the free sugars intake should be less than 5% of the total energy intake [32]. Polish nutritional standards [51] comply with the WHO [132] recommendations, that is, in the diets of children aged 1–18 years, the free sugars intake should be reduced to less than 10% of the total energy intake. Currently, the lower free sugars intake (below 5% of total energy intake) is considered difficult to achieve, but health benefits of such a significant reduction are not disputed [37].

– The role of **vitamins and minerals** in the body is related to its proper functioning in terms of structural, metabolic and regulatory processes, including those related to immunity [37, 112]. Vitamins and minerals should come from a variety of fresh and minimally processed foods due to their better bioavailability than from highly processed, reheated or long-stored foods, and their dietary sources should not be replaced with dietary supplements since their bioavailability from supplements is unknown [37, 137, 76, 70, 52]. It is believed that the vitamin D intake by the body should be additionally increased through supplementation, since this vitamin has multidirectional effects in the body, and there is high probability of insufficient intake of vitamin D in children’s diets and, consequently, the risk of its deficiencies in the body [98].

### 2.2 Nutrition of children in kindergartens and schools, and in other care and education facilities

Nutrition of children in kindergartens, schools or other care and education facilities is one of the elements supporting the children’s development and health, and may also have an educational function. Nutrition in such facilities should be based on proper nutrition guidelines described and/or presented in an accessible way, for example, as a healthy eating plate; it should fulfil the current nutritional standards and comply with the applicable legal regulations. Children are very sensitive to excess or deficiency of energy and nutrients, and therefore it is recommended that nutrition in kindergartens or other care and education facilities should be planned on the basis of RDA or AI, if there is no RDA for a given nutrient. For the energy value of the diet, this is the level of Estimated Energy Requirement (EER), because it is only at this level that recommendations for energy are specified. The use of a nutritional standard at the RDA level covers the demand for nutrients in almost all individuals/children in the group (97.5%) [51].

A typical preschool diet encompasses one to two main meals and one to two complementary meals, planned to provide approximately 75% of the daily energy intake and an adequate amount of nutrients. Breakfast should provide approximately 25% of the daily energy intake, second breakfast around 10%, lunch around 30% and afternoon snack around 10%, with the nutrient intake adjusted to the energy intake in the meal. A school diet usually includes one meal, that is, lunch, and in the case of children from the youngest grades, also a second breakfast. Lunch should provide 30–35% of the daily energy intake and be properly composed in terms of nutritional value and sensory quality. In mass catering, it is recommended to plan meals in a system for at least 10 days or longer, even monthly. This enables better planning of rational shopping, assessing whether the planned diet is not monotonous in terms of the selection of products, dishes, culinary techniques, and also establishing whether it is appropriately diversified and includes all product groups and appropriate proportions between them [133]. In educational facilities, local, seasonal and sustainably produced foods should be introduced as an element of promoting a diet that respects the environment. This allows us to prevent and reduce the amount of food waste in educational facilities through the use of practical solutions, which is an effective tool for environmental protection. Care should also be taken to ensure unlimited access to drinking water in schools/kindergartens and to promote water consumption, also during meals [*Stanowisko Komitetu Nauki o Żywieniu Człowieka Polskiej Akademii Nauk w sprawie posiłków szkolnych i nowych standardów żywienia w szkołach 2019*].

The solutions with respect to organising the nutrition of preschool and school-age children depend on the form of care and the time that the children spend in the facility. Meals prepared at home require special attention in the context of nutrition in kindergarten/school, for example, dinner or lunch and dinner in one for preschool children or breakfast, lunch and dinner for school children.

Since 2016, the amended Regulation of the Minister of Health on groups of foodstuffs intended for sale to children and adolescents in education system units and the requirements to be met by foodstuffs used in the collective feeding of children and adolescents in these units [103]. The provisions of the Regulation focus primarily on the proper selection of foods in relation to nutritional recommendations and the correction of the most common irregularities in the nutrition of children and adolescents, including reducing the consumption of foods with added sugars and sweeteners, foods high in fat, salt/sodium; and increasing the intake of vegetables, fruit and fish. According to the provisions of the Regulation, foodstuffs used in the collective feeding of children and adolescents must meet the appropriate requirements for a given age group, resulting from the current nutritional standards for the Polish population. They should be selected in such a way that the daily diet consists of food products from different food groups, which ensures appropriate diversity of the diet. Main meals (breakfast, lunch, dinner) must contain products from the following groups: grain products or potatoes, vegetables or fruits, milk or dairy products, meat, fish, eggs, fats and other products – nuts, pulses and other seeds.

Parents of children attending kindergartens/schools should be encouraged to get interested in the nutrition of their children outside the home. Home-made meals should complement the kindergarten/school diet, both in terms of nutrients and food products. This will allow for the proper balancing of children’s diets, including avoiding deficiency or excess of specific nutrients, which is often observed in both individual/home and collective nutrition [92].

### 2.3 Prevention of dental caries in the context of nutrition

Primary teeth and immature permanent teeth are particularly susceptible to the development of caries, and thus the period of increased susceptibility to caries begins with the occurrence of the first primary teeth and lasts until approximately 15–16 years of age. In the majority of children, permanent teeth begin to replace primary teeth at preschool age. Therefore, it is important to pay attention to adequate intake of nutrients influencing the structure of permanent teeth and to eating behaviours supporting the maintenance of health of primary teeth, since it was demonstrated that the presence of carious lesions in primary teeth is associated with a significant risk of the development of caries in permanent teeth [62].

The foods consumed may be a source of minerals necessary for the proper course of odontogenesis and remineralisation of enamel, and the diet may support self-cleaning of the oral cavity and stimulate the salivary glands to secrete saliva with a high content of compounds capable of buffering acids and maintaining the healthy condition of teeth and periodontal tissues.

Products supporting the maintenance of dental and periodontal health include unsweetened dairy products, especially hard rennet cheeses, which stimulate saliva secretion when chewed, contain also calcium and phosphorus necessary for the remineralisation of enamel, and the lipids present in those products create a protective film on the teeth surface against the activity of acids. It was proven that casein phosphopeptide forms a compound with calcium phosphate, increasing the plaque pH and constituting a reservoir of ions for enamel remineralisation [63]. For maintaining dental health, consumption of products containing arginine and dietary fibre was also found beneficial. Products containing fibre, in particular hard, raw vegetables and fruits, during their biting and chewing, mechanically clean the teeth and interdental spaces and stimulate saliva secretion, and the water they contain reduces the cariogenic effect of sugars present in those products. Dietary fibre has a beneficial impact on the composition of microbiota in oral cavity, thus reducing the formation of compounds leading to demineralisation of enamel [107]. Similarly, it is also beneficial to consume whole grain products, which not only contain dietary fibre, but are also a source of ingredients necessary for odontogenesis. In the diets of children, it is worth adding the products that are sources of proteins rich in arginine, which can beneficially modify the composition of the oral microbiota and is metabolized by some dental plaque bacteria, and the products of this transformation neutralize the acidic pH [33]. Arginine-rich foods include pumpkin seeds, sunflower seeds, sesame, nuts, beans, soybeans, lentils, rennet cheeses, beef and turkey meat.

Important factors influencing the process of self-cleaning of the oral cavity include the texture of the foods (non-sticky, hard, stimulating chewing), appropriate intervals between meals and the proper secretion of saliva, which washes the teeth and interdental spaces, thus cleaning and protecting the teeth. Appropriate salivary secretion depends on proper body hydration; therefore, drinking an appropriate amount of water should be one of the elements of anti-caries prevention. The reasons of unfavourable changes in the amount and composition of saliva may also include protein-energy malnutrition, deficiencies of vitamins A, C, D and of minerals – fluorine, calcium and phosphorus [61].

Cariogenic dietary ingredients include beverages with acidic pH and simple sugars, that is, glucose and fructose, as well as selected double sugars, mainly sucrose, which are transformed in the oral cavity into organic acids lowering the pH of dental plaque and contributing to enamel demineralisation, which begins when the pH of dental plaque falls to around 5.5. Free sugars have the highest cariogenic potential [132, 43]. Products containing both sucrose and starch (e.g. sweet cereals, pastries, cookies, wafers) were found to be more cariogenic than those containing only sucrose. This is due to the time needed to neutralize the acidic environment by saliva, which is much longer after the consumption of starch products with the addition of sucrose compared to the consumption of sucrose alone [100]. For the etiopathogenesis of dental caries, it is also important how often cariogenic products are consumed, what their texture is and when they are consumed. Foods with a sticky consistency and containing sugars remain on the teeth surface and in the interdental spaces for a long time. Therefore, frequent consumption of such products is conducive to constant acidity of dental plaque and to demineralisation of enamel, with limited possibility of remineralisation. It is, therefore, less harmful to eat such products once a day and to eat them during the main meal, when other dietary ingredients reduce their cariogenic impact and enable self-cleaning of the oral cavity. Particularly cariogenic products include sweetened carbonated drinks, fruit juices and drinks, which contain large amounts of free sugars (7–10 g/100 ml) and have a low pH due to the organic acids (mainly citric, malic, carbonic, and orthophosphoric acid) they contain.

For children, the best beverage to quench thirst is water. The amount of juices drank within a day should be limited and not exceed 150–200 ml, but children should be encouraged to eat fruits. Drinking sweet beverages and juices, and eating acidic vegetables and fruits between meals, should also be avoided.

Anti-caries prevention is supported by the recommendations of the World Health Organization to limit the consumption of free sugars to less than 10% of the energy value of the diet, which, with an energy intake of 1,400 kcal, corresponds to about 35 g, that is, about 7 teaspoons of sugar per day [132]. According to the latest recommendations, it is beneficial for overall and oral health to limit the consumption of free sugars to less than 5% of the energy value of the diet [4, 37, 32].

In children at particular risk of caries, it is recommended to partly replace sugar with xylitol, which has a sweet taste like sucrose but has anti-caries activity, since it is not metabolised by cariogenic bacteria and does not contribute to lowering the pH in the oral cavity [64]. Xylitol is added to many foods: chewing gums, syrups, milk, candies, jellies and other sweets. The American Academy of Pediatric Dentistry 11AAPD - American Academy of Pediatric Dentistry recommends using xylitol in children with moderate and high risk of dental caries [5]. The current AAPD guidelines do not specify the recommended dose of xylitol, but according to the position of the AAPD from 2014, the frequency of xylitol intake should be at least twice a day, and its amount should not exceed 8 g/day [3].

Summing up, caries prophylaxis in children should include regular consumption of meals; avoiding snacks (in particular sweet snacks); keeping adequate intervals between meals; eating foods with hard texture, stimulating chewing; avoiding mushy and sticky foods; eliminating carbonated and sweetened drinks from the diet and limiting the consumption of fruit juices in favour of eating fruit, drinking milk and still water; limiting the consumption of cariogenic foods (mainly sweets) in favour of less cariogenic and low-processed foods (vegetables, whole grain products, dairy products, nuts) [4, 88].

## 3. Nutritional education of preschool and early school-age children as a form of promoting healthy dietary behaviours and habits

Educational activities on health addressed to preschool and early school-age children form the basis for their healthy behaviours, including those related to proper nutrition [134]. In Polish children aged 11–12 years, evidence was found that multielement and activating nutritional education brings lasting effects even 9 months after its completion in terms of improvement of nutritional knowledge – a reduction in unhealthy dietary behaviours was found, which translated into a lower risk abdominal obesity, despite the concurrent increase in sedentary behaviours (screen time) [121, 123].

The preschool and early school period is the time when eating habits develop. Children’s eating behaviours and physical activity are influenced by both individual factors (heredity, age, gender) and environmental factors (family, peers, community and society). Changes taking place in the nervous system of preschool children are visible in the acquisition of new skills, for example, motor and language skills, by the children. Children’s ability to act purposefully and perform activities requiring accuracy and precision such as writing increases, and complex cognitive functions, such as thinking and reasoning, problem solving or decision-making, improve [75, 11]. Children’s cognitive development is also influenced by nutritional factors related to a properly balanced diet, including the intake of protein, polyunsaturated fatty acids, minerals, vitamins (nutrients of particular importance for the development of children’s nervous system are indicated in section 1.1) and antioxidants. Intestinal microbiota, adequate body hydration and duration and quality of sleep, as well as physical activity also play a significant role [45, 13]. The family, kindergarten and school play a very important role in stimulating children’s cognitive activity [136].

During this period, children become increasingly aware of their dietary preferences through direct contact with food, through tasting, smelling, touching and eye contact, as well as by observing the environment in which they grow up, for example, by observing the eating behaviours of other people. The preschool and early school period is also the time when the first conscious decisions regarding food choices are made and food preferences are formed. Eating behaviours developed early in life may translate into health status later on, and unfavourable ones are most often associated with obesity and other non-communicable diseases in adolescence and adulthood [26, 79, 39]. Therefore, educational activities undertaken since the youngest age should aim at developing and maintaining a healthy lifestyle in terms of the diet and physical exercise. Children at preschool and early school age are potentially susceptible group in the context of efficient nutritional education. The knowledge about psychoeducational factors modifying the eating behaviours in children, and appropriate nutritional education of both children and their caregivers may support the development of healthy eating habits [26]. It should also be remembered that the effectiveness of nutritional education may be boosted by combining the provided knowledge with practical activities, which, as showed by multicentre studies performed among preschool and school children, should constitute an important element in developing appropriate dietary habits in children [44, 69].

Table 5 presents the most common problems and the methods of correcting them.

**Table 5.** Parents’ and caregivers’ behaviours inhibiting the formation of appropriate nutritional habits in preschool and early school-age children [Daniels LA et al. 2019] [25]

**Parents’ and caregivers’ behaviours**	**Examples of behaviours**	**Outcome**
**Pressure**/coercion Verbally pressuring the child to eat a specific type of product, specific amount of product, or to eat within a specific time.	Specification of portions size and forcing the child to eat a specific portion Encouraging to eat “one more mouthful” Playing games leading to unconscious eating Offering reward in the form of attractive (liked) food for eating unattractive (not liked) food, e.g. dessert for eating vegetables Offering non-food reward for eating unattractive (not liked) food, e.g. watching a cartoon for eating vegetables Overriding self-feeding attempts in favour of feeding the child	The child is taught to eat for external reasons. The food intake is regulated by external and not internal factors (hunger, satiety). Increased risk of eating disorders.
**Conditions**/restriction Decisive and rigorous restriction of access to unhealthy foods.	Offering dessert only after the child eats the rest of the meal Strict prohibition of eating highly processed foods even though they are overtly available in the household Strict differentiation between “healthy” and “un- healthy” foods	Increased desirability of restricted food in the child. Increased risk of eating disorders.
**Rewarding with meals as a form of emotion regulation** Eating as means to regulate emotions.	Offering favourite goods, e.g. sweets, in return for good behaviour Offering food to calm the child, keep it quiet Praising the child for desired eating behaviour, e.g. eating the entire portion	Increased risk of emotional eating and other eating disorders in future.
**Lack of eating rules** Lack of clear rules on where and when the child eats. Lack of specific rules on the quality of consumed foods.	Variable and inconsistent meal frequency and location Young child is not included in family meals Child eats different food to the rest of the family No limits on range of foods from which the child can choose No structured eating conditions – eating when wandering around, e.g. during play, watching cartoons Distractions when eating, e.g., watching TV, access to toys	Reducing eating competences of the child. Restricting autonomous dietary choices of the child.
**Restriction of diversity** Feeding the child only with the foods and meals accepted and liked by the child.	Offering new food only once and not reoffering it, if the child does not like it Disguising new products and meals, manipulating the composition of meals Insisting/pressure on the child to eat foods that the child does not like Replacing products that are not liked with accepted ones Reward for eating not accepted food (instead of tasting) Force feeding	Reduced chance for forming appropriate eating habits in terms of proper balancing of meals.

### 3.1 Fussy eating

Fussy eating is a common phenomenon among preschool and early school-age children. It consists of the tendency to reject a large range of foods, including familiar foods eaten earlier and also eaten regularly by other family members. Unlike in the case of neophobia, which consists of avoiding trying new or unfamiliar foods, children who are fussy eaters may at first have a very varied diet, which becomes limited with time [67, 38].

The risk of fussy eating is influenced by the following factors:

- Prenatal factors - sensory experience *in utero*; maternal nutrition during pregnancy;
- Early postnatal experience - breast or formula feeding; weaning practices;
- Parental feeding practices - food choice, portion size, reinforcing strategies and strategies impairing the formation of proper nutritional habits, modelling;
- Environment - social, economic, presence of siblings; and
- Genetic factors – inherited behavioural traits and other innate neurobiological and physiological factors [38].

Fussy eating is a significant source of concern and anxiety of parents and caregivers, which may lead to tensions and conflicts in the relationship between the parent/caregiver and the child. In such circumstances, parents often apply practices which are not only ineffective in extending the food choices but deepen the problem further. Such practices include forcing the child to eat, rewarding and punishing, and negative emotional consequences on the part of the parent/caregiver when the child refuses to eat [67. Negative practices with respect to shaping dietary behaviours in preschool and early school-age children are presented in Table 5 [38]. Practices which facilitate the formation of proper dietary habits in children who are fussy eaters include:

- Exposure to varied foods;
- Social learning of dietary habits resulting, among others, from watching others during meals eaten together;
- Creating healthy dietary patterns and an appropriate food environment by adults (parents and caregivers); and
- Association learning resulting, among others, from positive associations of food with experience from the body and the emotions and atmosphere during the meal [55].

### 3.2 Exposure to food

Rejection and elimination of a specific food item from the diet is easier for a child than acceptance of a new product. Aversion can develop after only one experience of a specific food, while acceptance most often requires exposure to a specific product several times [55]. Children’s food preferences change as a result of experience, and therefore it is important to enable them to have a free contact with foods and meals. The research show that such strategy is more efficient than rewarding [7, 124]. Exposure to new foods is cumulative – the more new products and meals there are in the child’s diet, the faster they are accepted [79]. Another technique is to combine the sweet taste pleasant for the child with less accepted tastes. Furthermore, multisensory exposure is important, as it involves various senses: taste, smell, touch and sight in contact with a new food product. Research results suggest that multisensory exposures are effective in increasing the consumption of products not accepted by children [23].

Easy access to food also plays an important role. When food is easily available and ready to eat, children eat it more willingly. Among school children, fruit and vegetable intake is higher when these foods are provided in locations accessible to the child and in accessible sizes (e.g., an apple cut into wedges, a carrot cut into sticks) [95].

### 3.3 Social learning

Preschool and early school period is the time of intensive exploration of the world by the child, which is a result of numerous interactions with the surrounding environment, including the family, peers and school. Starting from the age of 3–4 years, eating is motivated not only by hunger but is also a response to environmental signals. This shows that various family and social factors affect the eating behaviours of children [95].

The key places where children may safely try varied foods and learn to appropriately compose meals are the family environment and the preschool or school facility [91].

a. Parental preferences

Children’s food-related knowledge, preferences and eventually consumption are closely related to parents’ preferences, beliefs and attitudes towards food. This results from the exposure and free access to foods that the parents like and eat. Parents’ beliefs about which foods are healthy and their own food experiences also affect children’s food choices [95]. A growing body of research shows similarities between parents’ food acceptance and preferences, and children’s consumption and willingness to try new foods. Mothers and children often show similar patterns of food acceptance and preferences. Children are more willing to try new foods after they have seen an adult eating the foods, and this is reinforced further if the adult is their mother. Children’s fruit and vegetables intake is also positively correlated with parents’ fruit and vegetables intake [95].

b. Parenting styles in forming eating habits in children

Feeding styles reflect the attitude of caregivers to maintaining or modifying the eating behaviours in children. Three styles are identified: authoritarian, permissive and authoritative. The authoritarian style consists of a rigid attempt to control the child’s eating, disregarding the child’s choices and preferences. An authoritarian parent forces the child to eat “healthy products” and does not allow to it “unhealthy products”. Permissive feeding is characterised by the lack of structure in feeding; the child can eat everything it wants, in any amount. The authoritative feeding model is a balance between authoritarian and permissive feeding. The child is encouraged to eat healthy foods but also has some choice of various food options [21, 118]. The parental style that is the most efficient in forming eating habits is the authoritative style, which encourages the child to eat varied, appropriately balanced meals, does not force specific eating behaviour, and at the same time, to a limited extent, corrects the inappropriate food choices [66].

Using food as a reward for performing a specific activity (e.g. sweets for tidying up toys) or for eating a specific food (e.g. dessert for eating the salad from lunch) has a negative impact on forming food preferences in children. Food used as a reward becomes increasingly attractive while food (or activity) to which the child is encouraged by eating loses its attractiveness. Such a strategy may temporarily increase the consumption of not-accepted foods, but in the long term, it leads to reduced preference for such foods and reinforces negative preferences [124].

It is important that children are not only passive recipients of meals prepared by their parents. As part of exposure to food, parents may encourage children to participate in preparing meals. Tasks entrusted to children should be adjusted to their age in order to further increase the sense of self-efficacy in children. It is worth encouraging parents to follow behaviours and practices increasing the children’s knowledge and skills with respect to meal preparation and composition. The following factors may efficiently encourage preschool and early school-age children to get involved in the preparation of meals:

- Reduction of barriers affecting the safety of food/meal preparation by preschool and early school-age children;
- Gradually increasing and expanding the options for the child to prepare meals on its own (e.g. washing vegetables, preparing batter for pancakes, peeling eggs, whipping egg whites, mixing ingredients for vegetable and/or fruit cocktails);
- Acquisition of meal preparation skills by children, (e.g. setting the table, washing dishes, participation in shopping);
- Developing parents’ skills of planning meals for children;
- Educating children about benefits of preparing meals together; and
- Supporting independence and sense of self-efficacy of children with respect to meal preparation [91].

c. Educational facility

Apart from the family environment, other important places promoting nutritional habits include kindergartens and schools, and in particular the nutritional education in those facilities, as well as the contact with and free observation of peers [59, 39]. The research on preschool and early school-age children demonstrated that mutual interaction and observation has a complex impact and can both increase and reduce the preferences and consumption of various products. This results, among others, from the atmosphere during meals eaten together and the peers’ reactions to the consumed foods 105]. However, the preschool and early school age is not a period (as is adolescence) where the influence of peers overrides the role of the family environment.

### 3.4 Structure and regularity of meals

Family meals have a significant impact on the eating habits of children. Children who have meals together with other family members eat more healthy foods and their diets are more valuable. It was found that family meals are positively correlated with a lower risk of obesity, higher frequency of consumption of fruits and vegetables, grain products and calcium-rich foods, as well as such nutrients as protein, calcium, iron, folic acid, dietary fibre and vitamins A, C, E and B6. In addition, family meals reduce the probability of skipping breakfast [24].

Meals should be served at regular times and at the family table. Combining meals with watching TV or using a tablet is an inappropriate practice. Such behaviours promote excessive consumption and effectively separate children from hunger and satiety signals. The research shows increased frequency and amounts of tasty and highly processed foods consumed by children who eat meals while watching TV, using a computer, a smartphone, etc. In comparison with children who do not use screens during meals, children from families where contact with electronic media takes place during mealtime consume fewer fruits, vegetables, and grains and more fast foods, snacks and soft drinks [66].

Parents should also take care about the atmosphere during meals. The situations perceived by children as negative and emotionally difficult reduce their willingness to eat and their food preferences. The meal-related situations perceived as positive lead to increased preferences of the child with respect to the consumed meal.

### 3.5 Media

Food marketing may be an important element influencing food preferences and choices among preschool and early school-age children, in particular if it involves a “brand hero”, that is, a character familiar to and positively associated by the child [56]. It was demonstrated that the advertisements of highly processed foods have a negative impact on food choices of children. At the same time, the promotion of healthy foods in various media, including books, publications, brochures dedicated to children could contribute to nutritional education of both caregivers and preschool and early school-age children [39].

## 4. Physical activity supporting psychomotor development of preschool and early school-age children

Physical activity, defined as any form of bodily movement produced by contractions of muscles, in which energy expenditure exceeds resting energy levels, is one of the basic human needs at every stage of development and a component of a healthy lifestyle. Its intensity depends on the period of life of an individual, and in each of these periods, it fulfils different functions. Physical activity is an extremely important factor influencing the proper somatic, mental and social development of children at developmental age [81, 135, 41, 129, 130]. In the first stage of the preschool period, the child’s skeleton is sensitive and flexible, and the curvature of the spine is not yet established, which means that poor body posture can easily develop if the distribution of movement and rest is uneven. Due to the weakness of these systems and the immaturity of muscles, a preschool-age child cannot endure long-term and monotonous physical effort. Therefore, parents and teachers should dose physical activity, respect the variability of body positions, and, above all, create opportunities for preschoolers to freely change the regular rhythm and pace of movements [99]. Apart from physical activities at kindergarten/school, children should take part in similar activities with peers outside the facility. Children at this stage of development are excessively mobile; therefore, physical activity should be focused on developing their manual skills, motor coordination, and reactions to acoustic and optical signals.

In children and adolescents, physical activity brings benefits in terms of improving physical fitness (cardiorespiratory and muscular fitness), cardiometabolic health (blood pressure, lipid and glucose blood levels, presence or absence of insulin resistance), bone health, cognitive outcomes (scores in learning), executive skills, mental health (by reducing symptoms of depression) and in terms of reducing the risk of obesity. Therefore, it is recommended that children and adolescents should be physically active for at least an hour a day on average throughout the whole week (moderate to vigorous physical activity, mainly aerobic). High-intensity aerobic exercises, as well as muscle- and bone-strengthening exercises, should be performed at least three days a week. In children and adolescents, sedentary behaviours (performed while sitting) are associated with negative health effects such as increased risk of obesity and poorer cardiometabolic health as well as reduced fitness, reduction of prosocial behaviours and shorter sleep duration. Therefore, it is recommended that children and adolescents limit the amount of time spent being sedentary, especially the amount of recreational screen time [30].

It is worth paying attention to the unfavourable, growing trend which is in opposition to physical activity and consists of increasing the time spent on sedentary activities by children and adolescents. Based on extensive evidence from the literature, the World Health Organization (130, 129, 18), for the first time, in order to mitigate health risks, announced specific recommendations not only on physical activity but also concerning a sedentary lifestyle, for the general population and for specific subpopulations. It was recommended that children and adolescents spend their recreational time on, for example, going for a walk or outdoor exercise, while the sedentary time in front of a computer, telephone or TV screen should be significantly limited. Developing specific skills and motor habits of children at preschool and early school age is the responsibility of parents with the support of the kindergarten/school [135, 97]. By creating the conditions for play and movement for preschool and school children, we contribute to their comprehensive development. The study conducted among over 30,000 children aged 4–18 years showed that each 10-minute positive difference in physical activity was associated with lower BMI and reduced cardiovascular risk [114]. The long-term study called CHOP, in which physical activity of a cohort of 600 children at 6, 8 and 11 years of age was assessed during the 5-year period, found a decrease in total physical activity by 75.3 minutes per day, in moderate/vigorous activity by 30.7 minutes per day – more pronounced in boys. The study showed a decline in physical activity and an increase in sedentary behaviours. Therefore, it was assumed that the age between 6 and 11 years is the crucial period for intervention to increase physical activity [106].

The analysis of the condition of various areas of physical activity of children in Poland, its determinants and physical fitness showed that the physical activity of children and youth in Poland is low, and this problem was further deepened by the Covid-19 pandemic [139]. The lack of recommended amount of physical activity may contribute to restricting certain processes in children’s development, increase the risk of motor disorders and postural defects, and inhibit the development of motor consciousness and activity in subsequent stages of life. A very frequent consequence of the lack of physical activity is also an increased body weight leading to overweight and obesity, which in turn are reasons for other ailments, such as hypertension, type 2 diabetes and osteoporosis [30]. Therefore, it is recommended that children and adolescents should limit the time spent in sedentary behaviours, especially recreational screen time [30].

The position statement was adopted unanimously, with two votes abstained.

## Notes

Position statement in Polish published in the journal Standardy Medyczne Pediatria in 2023: Halina Weker, Mariola Friedrich, Katarzyna Zabłocka-Słowińska, Joanna Sadowska, Anna Długosz, Jadwiga Hamułka, Jadwiga Charzewska, Piotr Socha, Lidia Wądołowska. Stanowisko Komitetu Nauki o Żywienia Człowieka Polskiej Akademii Nauk w sprawie zasad żywienia dzieci w wieku przedszkolnym (4–6 lat) i wczesnoszkolnym (7–9 lat). Position paper on nutrition of children aged 4–6 years and 7–9 years of the Committee of Human Nutrition Science of the Polish Academy of Sciences. Standardy Medyczne Pediatria. 2023 Sep–Oct;20(5):481–503.
